# Block vs. Traditional Periodization of HIT: Two Different Paths to Success for the World’s Best Cross-Country Skier

**DOI:** 10.3389/fphys.2019.00375

**Published:** 2019-04-05

**Authors:** Guro Strøm Solli, Espen Tønnessen, Øyvind Sandbakk

**Affiliations:** ^1^Department of Sports Science and Physical Education, Nord University, Bodø, Norway; ^2^Department of Neuromedicine and Movement Science, Centre for Elite Sports Research, Norwegian University of Science and Technology, Trondheim, Norway; ^3^Kristiania University College, Oslo, Norway

**Keywords:** endurance training, training intensity, intensity distribution, periodization model, block periodization, traditional periodization

## Abstract

In short-term studies, block periodization of high-intensity training (HIT) has been shown to be an effective strategy that enhances performance and related physiological factors. However, long-term studies and detailed investigations of macro, meso, and micro-periodization of HIT blocks in world-class endurance athletes are currently lacking. In a recent study, we showed that the world’s most successful cross-country (XC) skier used two different periodization models with success throughout her career. One including extensive use of HIT blocks, namely BP, and one using a traditional method namely TRAD. In this study, we compare BP with TRAD in two comparable successful seasons and provide a detailed description of the annual use of HIT blocks in BP. The participant is the most-decorated winter Olympian, with 8 Olympic gold medals, 18 world championship titles, and 114 world cup victories. Training data was categorized by training form (endurance, strength, and speed), intensity [low (LIT), moderate (MIT), and HIT], and mode (running, cycling, and skiing/roller skiing). No significant difference was found in the total endurance training load between BP and TRAD. However, training volume in BP was lower compared to TRAD (15 ± 6 vs. 18 ± 7 h/wk, *P* = 0.001), mainly explained by less LIT (13 ± 5 vs. 15 ± 5 h/wk, *P* = 0.004). Lower volume of MIT was also performed in BP compared to TRAD (13 vs. 38 sessions/year), whereas the amount of HIT was higher in BP (157 vs. 77 sessions/year). While BP included high amounts of HIT already from the first preparation period, followed by a reduction toward the competition period, TRAD had a progressive increase in HIT toward the competition period. In BP, the athlete performed seven HIT blocks, varying from 7 to 11 days, each including 8–13 HIT sessions. This study provides novel insights into successful utilization of two different periodization models in the worlds best XC skier, and illustrates the macro, meso and micro- periodization of HIT blocks to increase the overall amount of HIT.

## Introduction

Cross-country (XC) skiing is regarded as one of the most demanding endurance sports, with training and competition challenging every step of the oxygen transport chain. Thus, XC skiers’ training primarily targets the aerobic endurance capacity and the most common training model among XC skiers includes 700–850 h of endurance training, distributed as 90% low (LIT), 4–5% as moderate (MIT), and 5–8% as high-intensity training (HIT) (Sandbakk and Holmberg, 2017). Although HIT sessions normally make up only one to three of the weekly training sessions of XC skiers and many other endurance athletes, or ∼20% of the total annual number of sessions (Seiler, 2010; Stöggl and Sperlich, 2015), they are keys in eliciting physiological and performance gains (Laursen and Jenkins, 2002; Buchheit and Laursen, 2013a,b). In fact, it is argued that an increased volume and/or frequency of HIT would be beneficial for the further development of elite endurance athletes (Laursen, 2010).

Independent of the overall intensity distribution, most studies report that the periodization of HIT vs. MIT and LIT in XC-skiing is achieved via the traditional periodization model (Matwejew, 1975; Issurin, 2008; Tønnessen et al., 2014). Utilization of this model is characterized by mixed focus on LIT, MIT, and HIT in all periods, but with a gradual progression from high training volume to higher training intensity, reduced volume, and training that is more specific as the competition period approaches. However, the traditional periodization model has received criticism because of possible conflicting physiological adaptations produced by the mixed training of many performance-related factors simultaneously. As an alternative, it has been argued that a more effective way of organizing endurance training is to include defined blocks of increased focus on specific intensities (Issurin, 2008, 2010, 2016, 2018).

In this context, blocks of highly concentrated HIT stimulus aims to induce a beneficial metabolic impact and appropriate hormonal response to optimize the subsequent adaptations (Issurin, 2018). While positive short-term effects of using HIT blocks to augment training responses have been shown (Breil et al., 2010; Støren et al., 2012; Wahl et al., 2013; Clark et al., 2014; Rønnestad et al., 2017) only a small number of studies have compared block periodization of HIT with evenly distributed HIT-matched traditional-models. Some of these studies reported superior improvements by using HIT blocks among national-level cyclists and XC skiers (Rønnestad et al., 2014a,b, 2016), whereas a recent study of junior XC skiers reported no beneficial effects of blocking compared to an evenly distribution of HIT (McGawley et al., 2017). However, these studies have compared the different periodization models by matching the overall HIT stimulus, whereas the use of block periodization of HIT in a real-life context is often related to an increase in the overall HIT stimulus.

Although one long-term study followed a national level male cyclist through 58 weeks of systematic blocking of LIT, MIT, and HIT (Rønnestad and Hansen, 2018), most previous studies on block periodization of HIT are limited by short intervention periods (4–12 weeks), and none have examined endurance athletes at a world-class level. Furthermore, there is a lack of detailed investigations into macro-, meso-, and micro-periodization utilizing HIT blocks and evidence on how this model is distinguished from the traditional model according to the organization of training across the annual cycle in world-class endurance athletes.

In a recent study, we showed that the world’s most successful XC skier used two different periodization models with success throughout her career (Solli et al., 2017). One including extensive use of HIT blocks, namely BP, and a traditional method namely TRAD. In this follow-up case study, the main aims are to compare BP with TRAD in two comparably successful seasons in the world’s best XC skier, and to provide a detailed description of her annual use of HIT blocks. This will provide novel information on the macro-, meso-, and micro-organization of LIT, MIT, and HIT, and generate new hypotheses for follow-up studies.

## Materials and Methods

### Participant

The participant is the most-decorated winter Olympian, with 8 Olympic gold medals, 18 world championship titles, and 114 world cup victories (FIS, 2018). The study was evaluated by the regional ethics committee of mid-Norway and approved by the Norwegian Social Science Data Services (NSD). Written informed consent was obtained from the participant for publication of this study, which was performed according to the Helsinki declarations.

### Overall Design

This study builds on a previous longitudinal training study (Solli et al., 2017) identifying two training periodization models (block and traditional periodization of HIT) in the skier’s training patterns. Here, the detailed training content during one representative year using block periodization of HIT (BP: 2005–2006 season) and one representative year using the traditional model (TRAD: 2014–2015). The years was selected based on three criteria: (1) successful performance during the examined year (she won world cup races in both sprint (0.8–1.3 km) and distance (10–30 km) races in both seasons, which led to victory in the sprint and overall world cup), (2) equal endurance training load (ETL) based on training impulse (TRIMP), and (3) detailed information about the design of training sessions throughout the seasons.

### Monitoring, Registration, and Systematization of Training

The participant recorded all training data in diaries designed by the Norwegian Ski Association and the Norwegian Olympic Federation to provide a valid and accurate measurement of training (Sylta et al., 2014). All training data was systematized by phases [general preparation 1 and 2 (GP1 and GP2), specific preparation (SP), and competition phase (CP)], training form (endurance, strength, and speed), intensity (LIT, MIT, and HIT), and specific (skiing/roller skiing) vs. non-specific (running and cycling) exercise modes. Detailed information about the registration and systematization of training data, division of training phases, determination of intensity zones, categorization of LIT, MIT, and HIT sessions as well as the content of speed and strength sessions are previously described (Solli et al., 2017). Illness days and periods was registered based on the participant’s systematic reporting in the training diaries. ETL was calculated by multiplying the accumulated duration of the intensity by a multiplier for the particular intensity zone (e.g., 1 min at LIT, MIT, and HIT is given a score of 1, 2, and 3 TRIMP, respectively). Total ETL (TRIMP score) is then obtained by summating the results (Foster et al., 2001). The performance development throughout the season was investigated by comparing the average rank in international competitions (world cup races and the World/Olympic championships) in the first (i.e., races before the major championships) and the second phase (i.e., races from the major championships and throughout the rest of the competitive season). Since the average amount of MIT and HIT was 2–3 sessions per week (Solli et al., 2017), the definition of a HIT-block was set as >4 sessions of HIT (not including competitions) over a 7 days period.

### Interviews

To gather additional information, ensure compliance with the training diary commentaries, and verify the training intensity of different training sessions, both semi-structured interviews and specific questions regarding the experience of the two periodization models were conducted with the participant and her coaches.

### Statistical Analyses

All data from the investigated periods are presented as mean ± standard deviation (SD). Variables with normal distribution were analyzed by using a paired-sample *t*-test for BP vs. TRAD. Otherwise, the Wilcoxon signed-rank test was used. All statistical tests were processed using IBM SPSS statistics version 24 Software for Windows (SPSS Inc., Chicago, IL, United States) and Office Excel 2016 (Microsoft Corporation, Redmond, WA, United States).

## Results

### Comparisons of Block and Traditional Periodization of HIT

#### Total Training Volume and Load

**FIGURE 1:**
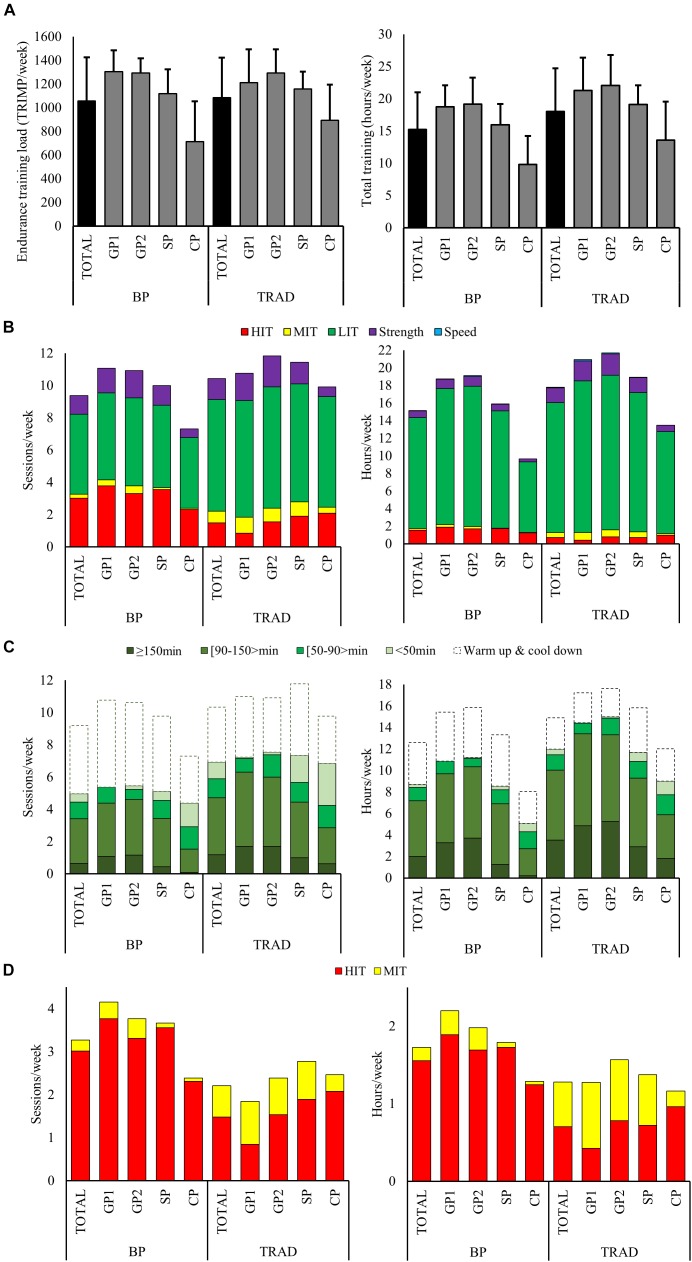
The distribution of the total training volume and endurance training load **(A)**, intensity distribution **(B)**, low (LIT) **(C)**, moderate (MIT), and high-intensity training (HIT) **(D)** across the annual cycle (TOTAL), and the different phases [general preparation (GP), specific preparation (SP) and competition phase (CP)], in the season using block periodization of HIT (BP) and the season using traditional periodization (TRAD).

No significant difference was found in the weekly ETL between BP and TRAD (1058 ± 368 vs. 1084 ± 339 TRIMP). Average weekly training volume was 15% lower in BP compared to TRAD (Table 1, *P* = 0.001). Total annual training volume was 795 h distributed across 478 sessions in BP and 938 h over 538 sessions in TRAD. The average weekly ETL and volume across the different phases are presented in Table 1 and Figure 1A.

**Table 1:** Weekly training distribution (mean ± SD) across the diffrent periodization phases in BP and TRAD for the world’s most successful female cross-country skier.

	GP1		GP2		SP		CP		Total	
	BP	TRAD	BP	TRAD	BP	TRAD	BP	TRAD	BP	TRAD
**Training forms**										
Hours	18.9 ± 3.4	21.3 ± 5.1	19.3 ± 4.0	22.1 ± 4.7^∗^	16.0 ± 3.2	19.1 ± 3.0	9.8 ± 4.4	13.6 ± 6.0	15.3 ± 5.9	18.0 ± 6.7^∗^
Session	10.8 ± 1.1	12.4 ± 2.0^∗^	10.6 ± 1.2	12.1 ± 1.0^∗^	9.8 ± 1.7	12.6 ± 1.3^∗^	7.3 ± 3.3	11.0 ± 2.9^∗^	9.2 ± 2.8	11.6 ± 2.3^∗^
**Training forms**										
Endurance (h)	17.6 ± 3.2	18.5 ± 4.6	17.9 ± 3.8	19.2 ± 3.9	15.1 ± 2.9	17.2 ± 2.6	9.3 ± 4.1	12.8 ± 4.9	14.4 ± 5.3	16.1 ± 5.5^∗^
Strength (h)	1.1 ± 0.5	2.3 ± 0.8^∗^	1.1 ± 0.5	2.4 ± 1.1^∗^	0.8 ± 0.3	1.7 ± 0.8^∗^	0.3 ± 0.4	0.7 ± 1.3	0.8 ± 0.6	1.7 ± 1.2^∗^
Speed (h)	0.2 ± 0.2	0.5 ± 0.4^∗^	0.3 ± 0.4	0.5 ± 0.3	0.1 ± 0.2	0.2 ± 0.2	0.2 ± 0.2	0.1 ± 0.1	0.2 ± 0.2	0.3 ± 0.3^∗^
**Exercise modes**										
Specific (h)	8.2 ± 2.6	9.9 ± 6.1	8.5 ± 4.2	9.7 ± 3.9	11.7 ± 3.0	12.7 ± 2.8	8.8 ± 3.8	10.8 ± 4.5	8.8 ± 3.7	10.0 ± 4.9
Non-specific (h)	9.6 ± 3.3	9.1 ± 3.0	9.7 ± 3.4	10.0 ± 2.8	3.6 ± 3.3	4.7 ± 2.5	0.7 ± 0.6	2.0 ± 1.9^∗^	5.7 ± 4.9	6.3 ± 4.1
SPE/UNSPE (%)	*46/54*	*52/48*	*47/53*	*49/51*	*77/23*	*73/27*	*92/8*	*84/16*	*61/39*	*61/39*
**Endurance training time**										
Load (TRIMP/wk)	1303 ± 183	1213 ± 282	1293 ± 124	1292 ± 202	1118 ± 207	1158 ± 148	712 ± 342	893 ± 302	1058 ± 368	1084 ± 339
LIT (h)	15.4 ± 4.1	17.2 ± 4.4	15.9 ± 4.6	17.6 ± 4.2	13.3 ± 2.7	15.8 ± 2.5	8.0 ± 3.4	11.6 ± 4.7	12.6 ± 5.2	14.8 ± 5.3^∗^
MIT (h)	0.3 ± 0.4	0.9 ± 0.6^∗^	0.3 ± 0.3	0.8 ± 0.7	0.1 ± 0.2	0.7 ± 0.6^∗^	0.04 ± 0.2	0.2 ± 0.3^∗^	0.2 ± 0.3	0.6 ± 0.6^∗^
HIT (h)	1.9 ± 1.5	0.4 ± 0.5^∗^	1.7 ± 1.1	0.8 ± 0.6^∗^	1.7 ± 0.5	0.7 ± 0.3^∗^	1.2 ± 1.0	1.0 ± 0.6	1.6 ± 1.2	0.7 ± 0.6^∗^
LIT/MIT/HIT (%)	*87/2/11*	*93/5/2*	*89/2/9*	*92/4/4*	*88/0/11*	*92/4/4*	*86/0/13*	*91/2/7*	*88/1/11*	*92/4/4*
**Endurance training sessions**										
LIT (sessions)	5.4 ± 3.3	7.2 ± 1.8	5.5 ± 3.1	7.5 ± 1.7	5.1 ± 1.5	7.3 ± 1.5^∗^	4.4 ± 2.3	7.2 ± 3.2	5.0 ± 2.6	7.0 ± 2.4^∗^
MIT (sessions)	0.4 ± 0.5	1.0 ± 0.7^∗^	0.5 ± 0.5	0.8 ± 0.7	0.1 ± 0.3	0.9 ± 0.9^∗^	0.1 ± 0.3	0.4 ± 0.5^∗^	0.3 ± 0.4	0.7 ± 0.7^∗^
HIT (sessions)	3.8 ± 3.0	0.8 ± 0.9^∗^	3.3 ± 2.3	1.5 ± 1.1^∗^	3.6 ± 1.0	1.9 ± 0.9^∗^	2.3 ± 1.5	2.1 ± 1.5	3.0 ± 2.2	1.5 ± 1.2^∗^
LIT/MIT/HIT (%)	*56/4/40*	*80/11/9*	*61/5/34*	*76/9/16*	*58/1/41*	*73/9/19*	*66/1/33*	*74/4/22*	*61/3/36*	*76/8/16*
**Categorization of low intensity training**										
Warm up & cool down (h)	4.6 ± 3.1	2.8 ± 0.5	4.6 ± 2.0	2.6 ± 1.1^∗^	4.8 ± 1.0	4.2 ± 1.2	3.0 ± 2.0	3.0 ± 1.9	3.9 ± 2.4	2.9 ± 1.4^∗^
<50 min (h)	0.0 ± 0.0	0.1 ± 0.2	0.1 ± 0.4	0.1 ± 0.3	0.3 ± 0.5	0.8 ± 0.9	0.8 ± 0.8	1.3 ± 1.5	0.3 ± 0.6	0.5 ± 1.0^∗^
50–90 min (h)	1.2 ± 1.3	1.0 ± 1.6	0.8 ± 0.8	1.5 ± 1.2^∗^	1.3 ± 1.7	1.5 ± 1.4	1.6 ± 1.3	1.9 ± 1.0	1.2 ± 1.2	1.4 ± 1.3
90–150 min (h)	6.4 ± 3.7	8.5 ± 4.0^∗^	6.7 ± 4.6	8.1 ± 3.0	5.7 ± 3.0	6.4 ± 2.9	2.5 ± 2.8	4.1 ± 3.9	5.2 ± 3.8	6.5 ± 3.9^∗^
≥150 min (h)	3.3 ± 3.0	4.9 ± 3.4	3.7 ± 3.6	5.3 ± 4.6	1.3 ± 2.0	2.9 ± 5.0	0.2 ± 0.7	1.8 ± 2.5^∗^	2.0 ± 2.9	3.5 ± 4.0^∗^

*GP1, general preparation period 1; GP2, general preparation period 2; SP, specific preparation period; CP, competition period; SPE, specific exercise mode; UNSPE, non-specific exercise mode; LIT, low intensity training; MIT, moderate intensity training; HIT, high intensity training. ^∗^Significantly different from BP (P < 0.05).*

The average rank in international competitions in the two investigated seasons was 4.2 ± 4.4 (2.9–5.6 in the first and second phase, respectively) in BP vs. 2.8 ± 5.8 (1.4– 5.8 in the first and second phase, respectively) in TRAD. Total annual days spent at altitude were 58 and 54 during BP and TRAD, respectively. The same number (36) of days without training were found in BP and TRAD. The number of illness days was seven (one period in CP) in BP compared to nine (one period in GP1, one period in GP2, and one in CP) in TRAD.

#### Distribution of Training Forms and Exercise Modes

The training forms in BP were distributed as 746 h (94%) endurance, 40 h (5%) strength, and 10 h (1%) speed training compared to 836 h (89%) endurance, 86 h (9%) strength, and 15 h (2%) speed training in TRAD. Weekly endurance-training volume was 11% lower in BP compared to TRAD (Table 1, *P* = 0.019). The weekly strength training time was 54% lower in BP vs. TRAD (Table 1, *P* < 0.001); this was present in all phases except CP. No difference was found in maximal strength training between BP and TRAD, but a much higher amount of general strength training (48 vs. 0.4 h) was performed during TRAD. The weekly speed training time was 37% lower in BP compared to TRAD (Table 1, *P* = 0.023). The distribution of training forms across phases is presented in Figure 1B.

The distribution of specific/non-specific exercise modes was approximately similar (61/39%) in BP and TRAD. No differences between the weekly amount of specific vs. non-specific training were found across the annual phases, except for 65% higher volume of non-specific exercise modes in CP during TRAD (Table 1, *P* = 0.016).

#### Endurance Training

According to the time spent in each intensity zone, the distribution of LIT/MIT/HIT was 88/1/11% in BP and 92/4/4% in TRAD. Quantified in terms of the number of sessions, this was 61/3/36% in BP and 76/8/16% in TRAD.

The volume of LIT was 15% lower in BP than TRAD (Table 1, *P* = 0.004). Further BP included 34% more LIT time performed as warm up or cool down in connection with MIT, HIT, or strength sessions (Table 1, *P* = 0.006), while 28% more of the LIT volume in TRAD was performed as sessions >90 min (Table 1, *P* = 0.002). The LIT time and sessions across the different categories of duration, in BP and TRAD are presented in Figure 1C.

A substantially lower number of MIT sessions was performed in BP than TRAD (13 vs. 38 sessions). Accordingly, weekly MIT time was 70% lower in BP than TRAD (Table 1, *P* < 0.001). The annual number of HIT sessions was much higher during BP (157 vs. 77 sessions), and weekly HIT time was 121% higher in BP compared to TRAD (Table 1, *P* < 0.001). The progression of MIT and HIT from GP1 to CP was different between BP and TRAD. While BP included high amounts of HIT already in the beginning of the annual training cycle, followed by a reduction toward CP, TRAD had a progressive increase of HIT toward CP. The distribution of MIT and HIT time and sessions, across the annual phases is presented in Figure 1D.

### Detailed Description of the Use of HIT Blocks in BP

#### Overall Training Load and Placement of HIT Blocks

**FIGURE 2:**
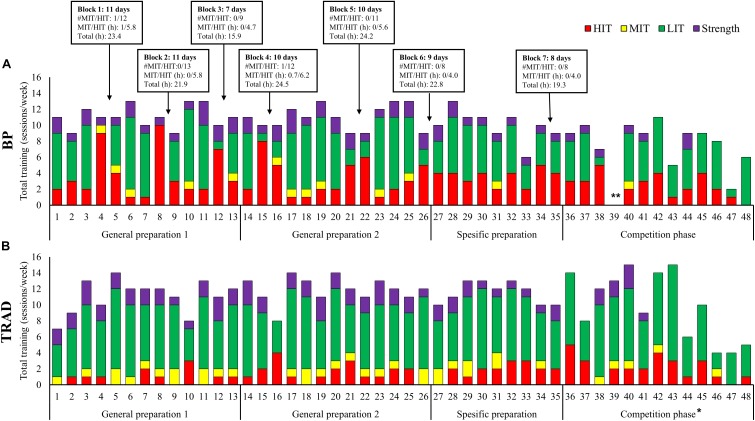
The placement of high-intensity training blocks and the weekly training sessions distributed into endurance training [low (LIT), moderate (MIT), high-intensity (HIT]) and strength training across the annual cycle using block periodization of HIT (BP) **(A)**, and the season using traditional periodization (TRAD) **(B)**. ^∗^The competition phase, includes many short duration LIT sessions (i.e., morning jogs or restitution sessions before and after competitions). ^∗∗^Week 39 in BP, contains no training since the participant was sick.

Seven HIT blocks with a duration of 7–11 days, including 8–13 HIT sessions, were performed during BP. A total of 35.6 h HIT was performed during HIT blocks representing 45% of annual HIT volume. Weekly ETL during HIT blocks was 1366 ± 68 TRIMP (Min-Max; 1254–1446 TRIMP). The average time between HIT blocks was 27 ± 12 days (15–48 days). The placement of HIT blocks and the weekly training content across the annual phases in BP compared to TRAD is presented in Figure 2A,B.

#### Distribution of Training Forms and Intensity

**FIGURE 3:**
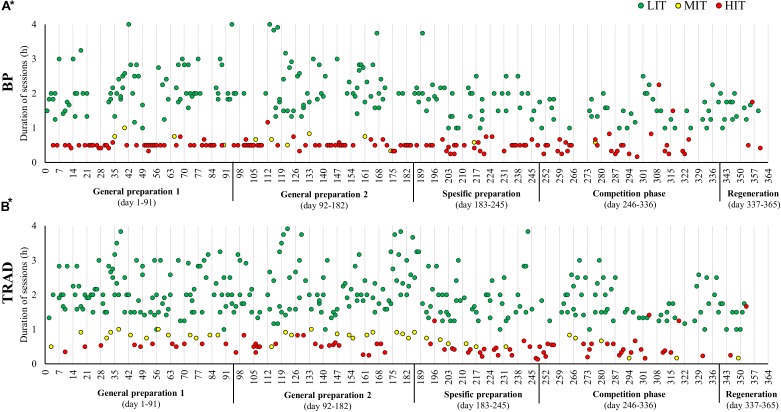
The duration of low (LIT), moderate (MIT), and high-intensity (HIT) sessions performed across the annual cycle using block periodization of HIT (BP) **(A)**, and the season using traditional periodization (TRAD) **(B)**. ^∗^LIT sessions with a duration < 1 h is not included in the figures.

Total training volume during HIT blocks was 16.2 ± 1.3 h/wk performed across 10.8 ± 0.4 sessions/wk. This included 15.1 ± 1.1 h/wk (93%) endurance training and 1.1 ± 0.4 h/wk (7%) strength training. Only 0.3 h of speed training was performed during one of the HIT blocks. Endurance training time was distributed into 11.1 ± 1.4 h/wk LIT, 0.2 ± 0.3 h/wk MIT, and 3.8 ± 0.5 h/wk HIT, which gives a time in zone distribution of 74/1/26% LIT/MIT/HIT. Weekly endurance training sessions were distributed as 1.3 ± 1.0 LIT, 0.2 ± 0.3 MIT, and 7.7 ± 0.9 HIT, which gives a session in zone distribution of 14/2/84% LIT/MIT/HIT. The total number and duration of LIT, MIT, and HIT sessions performed in BP and TRAD are presented in Figure 3A,B.

#### Exercise Modes and Design of Sessions

The 75 HIT (73 sessions) and MIT (2 sessions) sessions were distributed as 29% running, 17% running with poles, as well as 8% double poling, 22% skating, and 24% classic on snow or roller skiing. Eighty-seven percent of HIT sessions were performed as intervals with working periods of 4–7 min, 10% performed as continuous sessions and 3% performed as intervals < 4 min. The most typical HIT session was 5 × 4 min, with 2–3 min recovery between working periods (see typical examples of sessions in Table 2).

**Table 2:** Detailed description of the training performed during 7 days of one representative HIT block in BP and one high-load week in TRAD.

	Training content	
	**Block periodization**	**Traditional periodization**
1	AM: 2 h LIT, running on varying terrain	AM: 2.5 h LIT, classic on varied terrain, including sprints
	PM: 5 × 4 min HIT, classic roller skiing uphill terrain^∗^	PM: Warm-up 30 min + 1 h general and maximal strength training
2	AM: 5-4-5-4-5 min HIT, running with poles, uphill terrain^∗^	AM: 5 × 7 min MIT, skating on varied terrain^∗^
	PM: 6 × 4 min HIT, double poling, flat terrain^∗^	PM: 1.5 h LIT, running on varied terrain
3	AM: 5 × 4 min HIT, skating roller skiing uphill terrain^∗^	AM: 3 h LIT, 50/50% running and classic on varying terrain
	PM: Warm-up 30 min + 45 min maximal strength training	PM: 2 h LIT, skating on varied terrain, including sprints
4	AM: 4 × 6 min HIT, roller skiing alternating between classic and skating, uphill terrain^∗^	AM: 2 h LIT, running on varying terrain
	PM: Rest	PM: Warm-up 30 min + 1.5 h general and maximal strength training
5	AM: 5-4-5-4-5 min HIT, running flat terrain^∗^	AM: 2.5 h LIT, classic on varying terrain
	PM: 5 × 4 min HIT, skating roller skiing uphill terrain^∗^	PM: 1.5 h LIT, running on varied terrain including jumps and sprints
6	AM: 5-4-3-4-5 min HIT, running with poles, uphill terrain^∗^	AM: 6-5-4-4-4 min HIT, running with poles uphill terrain^∗^
	PM: Rest	PM: 1.5 h LIT, skating on varied terrain
7	PM: 6 × 4 min HIT, double poling, flat terrain^∗^ PM: Rest	AM: 2 h LIT, running on varying terrain PM: 2 h LIT, classic on flat terrain
TOTAL	• LIT (sessions/hours): 1/11.5 • MIT (sessions/hours): 0/0 • HIT (sessions/hours): 9/4.5 • Strength (sessions/hours): 1/0.75 • Speed (sessions/hours): 0/0 • Total (sessions/hours): 11/16.8 • Endurance training load (TRIMP): 1536 • Distribution of time (% LIT/MIT/HIT): 72/0/28 • Distribution of sessions (% LIT/MIT/HIT): 10/0/90	• LIT (sessions/hours): 10/22.8 • MIT (sessions/hours): 1/0.75 • HIT (sessions/hours): 1/0.6 • Strength (sessions/hours): 2/2.5 • Speed (sessions/hours): 3/0.75 • Total (sessions/h): 14/27.4 • Endurance training load (TRIMP): 1563 • Distribution of time (% LIT/MIT/HIT): 94/3/3 • Distribution of sessions (%LIT/MIT/HIT): 83/8/8

*LIT, low-intensity training, heart rate <87% max; MIT, moderate-intensity training, heart rate 87–92% max; HIT, high-intensity training, heart rate >92% max. ^∗^MIT and HIT sessions normally included 30–45 min of LIT as warm up and 15–30 min LIT as cool-down.*

The interviews revealed that the participant was very deliberate when performing HIT sessions during HIT blocks. This included a slow progression in workload allowing heart rate (HR) to gradually reach the target HR zone (>92% of HR_max_), keeping the pace steady when target HR was reached. A detailed description of the training performed in one high-load week during TRAD compared to 7 days of one representative HIT-block is presented in Table 2.

#### Training Between HIT Blocks

The average ETL between the HIT blocks was 1259, 1249, and 1087 TRIMP/wk during GP1, GP2, and SP, respectively. Total training volume was 20 h/wk during GP1-2 and 16 h/wk in SP, with the weekly average of HIT being ∼1 h and ∼2 sessions. According to training time, the intensity distribution of LIT/MIT/HIT was 93/1/6%, and this was 81/3/16% relative to the number of sessions. No HIT blocks were performed in CP.

The average ETL during the 7 days after each HIT block was 1198 TRIMP, with an average training volume of 19.7 h/wk including ∼7 LIT sessions, 1–2 MIT/HIT sessions, and 1–2 strength sessions.

## Discussion

In this study, we compared the use of block and traditional periodization of HIT, in two comparable successful seasons of the world’s best XC skier and provided a detailed description of macro, meso, and micro-organization of HIT blocks across an annual cycle. Despite equal ETL in BP and TRAD, a significantly higher training volume was found in TRAD, mainly due to longer duration of LIT sessions and more MIT. In contrast, twice as many HIT-sessions were performed during BP. The progression and distribution of HIT also differed between the 2 years: TRAD included a progressive increase in HIT toward CP, whereas BP included high amounts of HIT already in GP1, followed by a reduction toward CP. During BP, the athlete performed seven major HIT blocks, varying from 7 to 11 days with each including 8–13 HIT sessions. This contrasts with the organization of HIT in TRAD, in which only half the number of HIT sessions were performed, evenly distributed as 1–3 session/wk.

### Comparisons of Block and Traditional Periodization of HIT

This is the first study comparing successful utilization of block and traditional periodization of HIT in a world-class endurance athlete, where HIT blocks were used to significantly increase the amount of HIT. In this case the total ETL did not differ between the two investigated years, allowing for a valid comparison of the athlete’s macro-, meso-, and micro-organization of training volume, intensity distribution, content, as well as the design of training sessions.

A higher endurance training volume was found in TRAD, which was primarilly explained by more LIT (∼15 vs. 13 h/wk) including longer duration LIT sessions (∼5 vs. 3 sessions/wk > 90 min). More frequent exposure to longer duration LIT sessions in TRAD might have induced a positive long-term physiological adaptation that has also been highlighted in previous studies (Seiler, 2010). In BP, LIT was still a large proportion of the overall endurance training volume, but much of the LIT was performed as shorter bouts of warm up or cool down in connection with HIT sessions. Whether the total volume of LIT, independent of type of sessions, vs. a high volume due to longer duration LIT-sessions plays a role for the endurance adaptations is currently not known. As a compensation for the lower amount of LIT, as well as less MIT (13 vs. 38 sessions), in BP than TRAD, substantially more HIT training was performed (157 vs. 77 sessions), resulting in similar ETL across these two periodization models.

According to the time spent in each intensity zone, the distribution of LIT/MIT/HIT time was 92/4/4% in TRAD and 88/1/11% in BP, demonstrating a higher proportion of HIT in BP than previously reported in XC skiers (Tønnessen et al., 2014; Sandbakk et al., 2016; Sandbakk and Holmberg, 2017). Quantified in terms of the number of sessions, the intensity distribution in BP is further distinguished from TRAD and previous studies on XC skiing. In fact, the number of sessions at each intensity level shows a polarized distribution of 61/3/36% for LIT/MIT/HIT in BP. This number of HIT sessions in combination with the large annual training volume (∼800 h) is among the highest amount of HIT ever reported for elite endurance athletes in the scientific literature (Stöggl and Sperlich, 2015). This contrasts with the session distribution of 76/8/16% LIT/MIT/HIT observed in TRAD and differs substantially from the 20% distribution of HIT-sessions previously observed in elite endurance athletes (Seiler, 2010).

The major portion of HIT sessions in BP were performed as interval sessions with relatively short work duration (typically 5 × 4 min > 92% of HF_max_), while more of the longer duration MIT sessions (typically 5 × 7–8 min @ 87–92%) were employed in TRAD. Physiological adaptations depend on both intensity and accumulated duration of training. For example, accumulating ∼30–45 min at ∼90% HR_max_ twice per week has been found to be more effective than accumulating 15–20 min at ∼95% HR_max_ (Sandbakk et al., 2013; Seiler et al., 2013). This indicates that, in our athlete who performed MIT sessions at around 90% of HR_max_, the effects of the higher number of HIT sessions in BP could have been compensated for by a higher number of long-duration MIT sessions in TRAD.

We found opposite patterns in the distribution and progression of MIT and HIT across the phases of the training year in the two periodization models. In BP, the amount of HIT was already high in GP1 (1.9 h/wk) with a small reduction in SP (1.7 h/wk) and a further reduction in CP (1.3 h/wk). There was also a tendency for the HIT blocks to become shorter with fewer HIT sessions toward CP. In contrast, the amount of HIT was lowest in GP1 (0.4 h/wk) in TRAD, then increased toward CP (1.2 h/wk), whereas the amount of MIT showed the opposite pattern. Thus, TRAD represents a gradual change to a more polarized distribution with increased amounts of HIT closer to the desired peak performance. This is consistent with previous observations in world class XC skiers and orienteers (Tønnessen et al., 2014, 2015).

The different progression in HIT clearly represents diverse training philosophies. Interviews with the participant and her coaches indicate that the HIT blocks were aimed at enhancing VO_2max_ early in the preparation phase. This in order to facilitate higher training velocities throughout GP and SP and thereby be at a higher performance level as the CP approached. This is in contrast to TRAD, in which high volumes of LIT during GP are believed to provide an aerobic platform on which to build specific adaptations in response to increased HIT and optimization of performance toward CP (Laursen, 2010).

Despite clearly different volumes and organization of HIT, we found no difference in the performance development from the first to the second phase of the competition season within BP compared to TRAD. However, a limitation of our comparison of performance in BP vs. TRAD is that the traditional model was utilized later in the athlete’s career, when she was more experienced and that the competition program in XC-skiing changed during the investigated period. Further studies are therefore needed to investigate performance outcomes of different HIT-progression across the general and specific preparation phases.

### Detailed Description of the Use of HIT Blocks in BP

The present study is the first to give detailed insights into successful use of HIT blocks across an annual cycle in a world class endurance athlete. Of the 157 HIT sessions performed in BP, 73 sessions were organized during the HIT blocks, representing ∼45% of annual HIT volume and sessions. Specifically, seven HIT blocks, with a duration of 7–11 days including 8–13 HIT sessions, were performed from GP1 to SP. With such a high density of HIT sessions, a challenge is the short recovery time and a subsequent risk of accumulation of fatigue from sessions targeting the same systems. This challenge was indeed the case for our participant, where the average frequency of HIT sessions was ∼8 sessions over a 7 days period, with 1–3 days that included two HIT sessions performed on the same day. Although gradual fatigue over the period was sometimes the case, interviews with the participant indicate that she was mostly able to maintain a relatively high training quality throughout the HIT blocks, including when two HIT sessions were performed on the same day. In contrast, Yeo et al. (2008) reported the power output produced by endurance-trained athletes to be lower during a second session of HIT performed on the same day compared to a separate day. Reduced HR during maximal exercise has also been demonstrated in competitive cyclists following a period of training predominantly consisting of HIT (Jeukendrup et al., 1992). However, in the study by McGawley et al. (2017), the authors found no difference in either time in HR zones or distance covered resulting from performing nine vs. three HIT sessions a week.

An important point in this context is that XC skiers alternate between several exercise modes in their training. During HIT blocks, the participant utilized five different exercise modes including specific (classic and skating technique on skis or roller-skis), semi-specific (double poling or running with poles), and non-specific (running) exercise modes. This micro periodization of different exercise modes, with differential loading of the upper and lower body, is likely very important in maintaining the quality of sessions as well as avoiding muscular fatigue throughout the HIT blocks. Furthermore, both the participant and her coaches reported that the HIT sessions during blocks were closely supervised with a focus on a progressive increase in speed from the start of the sessions allowing HR to gradually approach the target intensity zone without increasing the velocity further when target HR (>92% of HR_max_) was reached. Her coaches stated that “after completing the last interval of a HIT session, the athlete should potentially be able to perform one additional interval at approximately the same pace.” This precise steering of intensity is critical, and differs from studies that investigate HIT protocols when performing HIT sessions at the maximum sustainable intensity during all interval bouts, so-called “isoeffort” matching (Sylta et al., 2016). In the current case, the precise steering of intensity during HIT sessions likely contributed to a reduced recovery time, thereby increasing the participant’s ability to perform the high frequency of HIT sessions at high quality and tolerate the overall training load during blocks.

Only a minor difference in the number of illness days or periods was found between BP and TRAD in our study. However, intensified training or frequent competitions have previously been associated with negative changes in immunological variables and more illness incidents (Tiollier et al., 2005; Papacosta and Nassis, 2011; Li et al., 2012; Svendsen et al., 2015, 2016). Another concern regarding the use of HIT blocks is the reduction in well-being and increased stress levels (Jeukendrup et al., 1992; Halson et al., 2002; Jurimae et al., 2004; Coutts et al., 2007). Therefore, the high stress induced by HIT blocks might increase the risk of long-term performance decline, overtraining, or non-functional over-reaching when stress and recovery is not sufficiently balanced (Meeusen et al., 2013). To prevent this, our participant had a reduction in training load after each HIT block and clearly reduced the amount of HIT sessions between blocks. We speculate that this was the main reason for the low number of sickness days, as well as her high motivation for training in both seasons analyzed.

The participant had progressively increased her training load since junior level and was in a highly trained state when she started the relatively extreme use of HIT blocks presented here (Solli et al., 2017). This, as well as her balanced micro-periodization of HIT blocks, made her able to tolerate high loads of HIT, increasing her performance rapidly during the first 2–3 years utilizing this periodization model. However, it should be noted that her performance stagnated during the following years. In retrospect, both the participant and her coaches agree that block periodization of HIT was effective during the first seasons (2004–2006), but that they subsequently should have changed the training to focus more on maintaining the increased capacity and targeting other performance-related factors. This coincides with the participant’s next major performance improvement in 2010 (Solli et al., 2017), including a change to a traditional model presented in the current study. Therefore, it could be stated that block periodization of HIT is effective in inducing rapid performance improvements but has some limitations and risks in respect to long-term utilization.

### Limitations

The main limitation of this study is that BP and TRAD were carried out with almost 10 years between them, so that effects of training history might differ between the two periodization models examined for this specific athlete. Although the ETL was similar in the two investigated years, differences in content, intensity distribution, duration, and frequency of sessions clearly differed. When interpreting our results, it is hence important to be aware of the integrated effects of both inclusion of HIT blocks and differences in overall training content. In addition, the development of XC-skiing and changes in the competition program makes it difficult to directly compare the performance development throughout the investigated years. Unfortunately, no test data was available for BP that could have allowed more detailed analyses of the development of performance indicators throughout the annual cycle. Furthermore, the main focus in this study was to compare the training organization and content between BP and TRAD and other components important for the training outcome such as physical characteristics, abilities, mentality, lifestyle and nutrition are not discussed here. Still, this study provides unique insights into how relatively extreme HIT blocks are managed in a world-class athlete, and how this differs for the same athlete using a traditional model.

### Practical Applications

This study shows that block periodization of HIT was successfully utilized in a world class XC-skier. In particular, we highlight the importance of balanced micro-periodization during HIT blocks by utilizing different exercise modes, careful steering of intensity, and reductions in the training load and amount of HIT after each block. In addition, the periodization model must be adjusted to the athlete’s training status, and the risk of negative over-reaching and stress on the immunological system must be considered. However, the participant also achieved substantial success using a traditional model, which might be considered a “safer” model. We hope that our study can highlight the importance of tailoring training to each individual athlete based on training history and other factors influencing adaptation to training.

## Conclusion

This study provides novel insights into successful utilization of two different periodization models by the world-leading XC skier of our time. Despite similar ETL, a higher training volume due to more MIT and long duration LIT sessions was found in TRAD. In contrast, twice as many HIT-sessions were performed in BP, which is among the highest volumes of HIT ever reported for elite endurance athletes. This high HIT volume was achieved by organizing 45% of the annual HIT sessions across seven HIT blocks, varying from 7 to 11 days, with each block including 8–13 HIT sessions. The progression and distribution of HIT differed clearly between periodization models: BP included high amounts of HIT already from the first preparation period, followed by a reduction toward CP, while TRAD had a progressive increase in HIT toward the CP. Altogether, this study illustrates two successful ways of periodizing endurance training in a world class athlete.

## Ethics Statement

The study was evaluated by the regional ethics committee of mid-Norway and approved by the Norwegian Social Science Data Services (NSD). Written informed consent was obtained from the participant for publication of this study, which was performed according to the Helsinki declarations.

## Author Contributions

GS performed data collection and performed data and statistical analysis. GS, ET, and ØS designed the study, contributed to interpretation of the results, wrote the draft manuscript, and contributed to the final manuscript.

## Conflict of Interest Statement

The authors declare that the research was conducted in the absence of any commercial or financial relationships that could be construed as a potential conflict of interest.
